# Long-term follow-up of Chinese patients with methylmalonic acidemia of the cblC and mut subtypes

**DOI:** 10.1038/s41390-024-03581-x

**Published:** 2024-09-21

**Authors:** Lili Hao, Shiying Ling, Si Ding, Wenjuan Qiu, Huiwen Zhang, Kaichuang Zhang, Ting Chen, Xuefan Gu, Lili Liang, Lianshu Han

**Affiliations:** Department of Pediatric Endocrinology and Genetic Metabolism, Xinhua Hospital, Shanghai Institute for Pediatric Research, Shanghai Jiaotong University School of Medicine, Shanghai, China

## Abstract

**Background:**

Methylmalonic acidemia (MMA) is the most common organic acidemia in China, with cblC (cblC-MMA) and mut (mut-MMA) being the predominant subtypes. The present study aimed to investigate the prognostic manifestations and their possible influence in patients with these two subtypes.

**Methods:**

A national multicenter retrospective study of patients with cblC-MMA and mut-MMA between 2004 and 2022 was performed. We compared the clinical features between patients with two subtypes or diagnosed with or without newborn screening (NBS) and further explored the potentially influential factors on the prognosis.

**Results:**

The 1617 enrolled MMA patients included 81.6% cblC-MMA patients and 18.4% mut-MMA patients, with an overall poor prognosis rate of 71.9%. These two subtypes of patients showed great differences in poor prognostic manifestations. The role of NBS in better outcomes was more pronounced in cblC-MMA patients. Predictors of outcomes are “pre-treatment onset”, “NBS”, variants of c.80A > G and c.482G > A and baseline levels of propionylcarnitine and homocysteine for cblC-MMA; “pre-treatment onset”, “responsive to vitB12”, variants of c.914T > C and baseline propionylcarnitine and propionylcarnitine/acetylcarnitine ratio for mut-MMA. Besides, prognostic biochemical indicators have diagnostic value for poor outcomes in mut-MMA.

**Conclusions:**

The study provided potential predictors of the long-term outcome of patients with cblC-MMA and mut-MMA.

**Impact:**

- Predictors of outcomes are “pre-treatment onset”, “NBS”, *MMACHC* variants of c.80A > G and c.482G > A and baseline propionylcarnitine and homocysteine for cblC-MMA, “pre-treatment onset”, “responsive to vitB12”, *MMUT* variants of c.914T > C and baseline propionylcarnitine and propionylcarnitine/acetylcarnitine ratio for mut-MMA.
- This study with larger sample sizes effectively validated the prediction power and emphasized the importance of NBS in improving the outcomes of both MMA subtypes.
- The study enhances understanding of the phenotypic and prognostic variations of MMA disease and the predictors will help in the improvement of diagnosis and treatment strategies to achieve a better prognosis for MMA.

## Introduction

Methylmalonic acidemias (MMA) comprise rare inborn metabolic errors that are mainly inherited in autosomal recessive mode.^1,2^ The global average incidence of MMA is about 1:100,000 and ranges from 1:3500 to 1:39,000 in China.^3,4^ MMA is caused by disorders of methylmalonyl-CoA mutase (MCM) or cobalamin metabolism and is characterized by the abnormal accumulation of propionylcarnitine (C3), methylmalonic acid, methylcitrate (MCA) and other metabolites, which often leads to multiorgan damage.^5,6^ The clinical phenotypes of MMA are variable and atypical, with onset ranging from prenatal to adulthood and clinical symptoms ranging from minor to life-threatening.^7,8^

According to whether homocysteinemia (Hcy) is combined biochemically, MMA can be divided into combined and isolated types.^1^ In China, 60%–80% of MMA cases are combined, with the cblC subtype (caused by *MMACHC* gene mutations, cblC-MMA) predominant (95%) and *MMUT* gene mutations (mut-MMA) cause 90% of the isolated MMA population.^9,10^ Both subtypes share some clinical symptoms including feeding difficulties, hypotonia, failure to thrive, acidosis, anemia and cognitive impairments.^1, 6,11,12^ However, mut-MMA cases are more likely to experience more serious clinical manifestations like acute decompensation and metabolic stroke, chronic kidney disease, and the urine MMA (uMMA) level is higher than that of cblC-MMA children, whereas cblC-MMA patients are more susceptible to hydrocephalus and microangiopathy-related manifestations (i.e., atypical hemolytic uremic syndrome), and retinopathy.^13–19^ However, comparative studies of the long-term prognosis between these two subtypes were rarely seen.

The current standard therapy for MMA patients depends on vitamin B12 (VitB12) responsiveness. It has been reported that cblC-MMA are responsive, requiring l-carnitine, betaine, folic acid and intramuscular injections of VitB12, while most mut-MMA patients are VitB12 unresponsive and need a protein-restricted diet and l-carnitine.^20–22^ The tandem mass spectrometry (MS/MS)-based newborn screening (NBS), facilitating early diagnosis and treatment initiation, appeared to have protective effects on the MMA prognosis, especially for the cblC subtype.^6, 23, 24^ Nevertheless, the prognostic benefit of NBS for the mut-MMA remains controversial.^25^ In addition, despite improved screening and treatment, some patients still face poor long-term outcomes, including intellectual disabilities, movement disorders, language impairment, ocular complications and even death.^6, 23, 26, 27^ The long-term outcome for MMA individuals in China remains to be elucidated.

In this national multicenter retrospective study, based on the medical records of a large MMA cohort, we analyzed clinical features and the influence of NBS on two MMA subtypes. Further, we investigated the impact of different factors on disease prognosis.

## Methods

### Patients

A total of 1617 MMA patients who underwent genetic analysis were diagnosed and treated at multiple Chinese hospitals from January 2004 to December 2022. Among them, 1319 cases were caused by the *MMACHC* gene variations and 298 cases were caused by the *MMUT* gene variations. All of these patients were followed up at least once and included in our study. This study has been in accordance with the Helsinki Declaration and was approved by the Ethics Committee of Xinhua Hospital, Shanghai Jiaotong University School of Medicine (Approval ID: XHEC-D-2023-142).

### Biochemical examination

Dried blood spots (DBSs) were collected and the levels of amino acids, free carnitine, and acylcarnitines were analyzed using API4000/API4500 MS/MS (Applied Biosystems, Foster City, CA). Urinary organic acids including methylmalonic acid (uMMA) and methylcitric acid (uMCA) were measured by QP2010/QP2020 gas chromatography–mass spectrometry (GC–MS, Shimadzu Limited, Kyoto, Japan). Plasma Hcy was detected using a fluorescence polarization immunoassay. Furthermore, routine blood and urine tests were performed to assess liver, renal and cardiac function. In the study, the baseline levels of characteristic metabolites are from the test results at the time of diagnosis; the levels after treatment are from the best test result during the follow-up.

### Genetic testing

Genomic DNA was extracted from peripheral blood using Qiagen Blood DNA Mini Kits (Qiagen, Hilden, Germany). Sanger sequencing or next-generation sequencing were performed and variations were identified using reference sequences from Genbank (*MMACHC*: NM_015506; *MMUT*: NC_000006.12). For the variants that were not recorded in the Human Gene Mutation Database, the ClinVar Database (https://www.ncbi.nlm.nih.gov/clinvar/) or previous literature, the pathogenicity analysis of these variants was performed according to the American College of Medical Genetics and Genomics and the Association for Molecular Pathology (ACMG-AMP) guidelines.^28^ Patients with other variant genes associated with MMA were excluded from this study.

### Treatment

Totally 1418 patients underwent a VitB12 loading test as previously reported.^9^ A reduction of over 50% in the blood C3/acetylcarnitine (C2) ratio or uMMA level following VitB12 treatment was regarded as complete responsiveness.^22^ A decrease of 50%–30% was considered partially responsive.^29^ Other cases were considered VitB12 unresponsive. In this study, 1295 patients (1189 with cblC-MMA and 106 with mut-MMA) who were completely or partially responsive to VitB12 treatment were regarded as VitB12 responsive.

The cblC-MMA and VitB12 complete responsive mut-MMA patients received treatment with hydroxocobalamin (1–20 mg, once every 1–20 days based on age, weight, and condition, intramuscular injection), levocarnitine (50–100 mg/kg/day, oral), betaine (50–100 mg/day, oral) and folinic acid (5–15 mg/day, oral). VitB12 partially responsive mut-MMA patients were treated with hydroxocobalamin (1 mg, once every 2–7 days), levocarnitine, and a protein-restricted diet limiting isoleucine, valine, threonine and methionine. Unresponsive cases were restricted to natural proteins, supplemented with a specially formulated nutritional powder and received levocarnitine. Blood amino acid and carnitine profiles, especially branched-chain and essential amino acids, were monitored to guide diet and drug therapy.^30–32^ Additionally, four mut-MMA patients underwent liver transplants.

### NBS

DBSs were taken 72–120 h after birth and were immediately sent for MS/MS examination.^33^ Newborns with positive results (C3/C2 > 0.2 and/or C3 > 4.0 μmol/L) in the initial screening were recalled and re-examined. The still-positive individuals were referred for confirmatory tests including GC–MS (uMMA and uMCA), genetic testing and examination of their Hcy level. Patients who underwent NBS (including those who developed symptoms during the screening process) were enrolled in the NBS group, and all patients were treated immediately after diagnosis. Patients who refused to receive NBS, developed disease before NBS, or were confirmed by NBS but refused treatment were included in the non-NBS group.

### Follow-up and prognostic evaluation

MMA patients were followed monthly initially, later extending to 3 months when stable. Follow-up methods included telephone surveys and in-person patient visits. During telephone surveys, parents supplied information on their children’s height, weight, motor, speech and cognitive development during telephone surveys. During follow-up visits, outpatients were evaluated by experienced pediatric clinicians.

The prognosis was evaluated according to the biochemical results and development status, which were categorized as poor or good. A good prognosis means no onset or significant abnormalities at the last visit. Poor outcomes included neurologic disorders, ocular complications, renal diseases, anemia, death and other abnormalities not individually counted (heart lesions, etc.).^30^ “Ocular complications” manifest as fixate inability, strabismus, nystagmus, and various ocular abnormalities such as maculopathy, retinopathy and optic atrophy. “Nephropathy” was primarily diagnosed through urine routine, renal function tests and renal ultrasound. “Anemia” mainly relied on routine blood tests. The “neurologic disorders” were based on the basic motor and language function assessed as previously described,^23, 34^ neuropsychological tests and cranial magnetic resonance imaging (MRI). The developmental quotient and intelligence quotient determined by neuropsychological tests followed a reported protocol.^9^ MRI abnormalities included hydrocephalus (ventriculomegaly, severe cerebral parenchyma atrophy and subdural effusion), periventricular white matter changes, corpus callosal thinning, myelination delay and basal ganglionic abnormalities.

### Statistical analysis

SPSS 26.0 (SPSS Inc., Chicago, IL) and GraphPad Prism Software 7.0 (Dotmatics, Boston, MA) were used for statistical analyses. Analyses using the Shapiro–Wilk method indicated a non-normal distribution for continuous variables, so the values are expressed as the interquartile range (IQR) and the differences between two groups were analyzed by a two-tailed unpaired or paired Student’s *t*-test with a Mann–Whitney *U* test. The differences between three or more groups were analyzed using Kruskal–Wallis one-way ANOVA and the post hoc Tukey–Kramer test. The comparison of rates used a two-tailed Fisher’s exact test with or without the Benjamini–Hochberg correction. A *p* value of <0.05 was considered statistically significant.

## Results

### Demographics and clinical features of enrolled patients

A total of 1617 MMA patients (900 males and 717 females) were enrolled in this study, which included 1319 (81.6%) cblC-MMA cases and 298 (18.4%) mut-MMA cases. Demographic, clinical and biochemical data are presented in Tables 1, 2 and Fig. S1. Among them, 1330 patients experienced onset, from minutes after birth to 32.3 years, and there are 21 cases in which the onset of symptoms was unknown. A total of 1586 patients provided information on whether they participated in the extended NBS program. Of the 670 patients who were diagnosed through NBS, 252 were asymptomatic and 206 experienced onsets before treatment (Fig. S1). Of the 916 patients who were not screened, 899 (98.11%) were diagnosed symptomatically, and 20 (21.8%) were confirmed via sibling diagnosis, including 13 asymptomatic patients (Fig. S1). Among the 1418 patients with treatment information, 1295 (91.3%) responded to VitB12 treatment, 942 of the 1220 patients (77.2%) had developed disease prior to treatment and 198 patients lost treatment onset information (Table 1, Fig. S1). All patients were followed up at least once, of whom 455 (28.1%) had normal development, while 1162 (71.9%) had poor outcomes, of which 135 (11.6%) were deceased. The median follow-up period was 24.1 months (IQR = 11.3–46.8 months). The overall levels of characteristic metabolites (blood C3, C3/C2 ratio, Hcy, uMMA and uMCA) before and after treatment are presented in Table 2, which shows that all indicators have improved after treatment.

**Table 1:** Comparison of clinical characteristics between cblC-MMA and mut-MMA patients.

Characteristics	Overall	cblC-MMA	Mut-MMA	*p* value
No.	1617	1319	298	
Male sex, No. (%)	900 (55.7)	732 (55.5)	168 (56.4)	0.783
Age during follow-up (years), median (IQR)	2.46 (1.11–5.01)	2.48 (1.13–5.01)	2.32 (1.08–5.03)	0.556
**Onset of disease**				
Onset age (months)	1.68 (0.24–6.9)	1.87 (0.36–6)	0.42 (0.12–10.59)	0.0242
Onset of symptoms, No. (%)^a^	1330 (83.4)	1084 (83.3)	247 (83.7)	0.865
Early onset, No. (%)^a^	923 (83.1)	745 (83.2)	178 (82.4)	0.77
**Diagnosis**				
NBS, No. (%)^a^	670 (42.2)	558 (42.9)	112 (39.2)	0.224
**Treatment**				
Interval from onset to treatment (days)	29 (6–96)	31 (8–105)	9 (2–38)	<0.0001
Pre-treatment onset, No. (%)^a^	1106 (79.2)	900 (79.3)	206 (78.9)	0.895
Responsive to vitB12 treatment, No. (%)^a^	1295 (91.3)	1189 (100)	106 (46.3)	<0.0001
**Outcome**				
Good prognosis, No. (%)	455 (28.1)	371 (28.1)	84 (28.2)	0.983
Poor, No. (%)	1161 (71.8)	948 (71.9)	214 (71.8)	
Deceased, No. (%)^b^	135 (11.6)	63 (6.6)	72 (33.8)	<0.0001
Ocular complications, No. (%)^a,b^	108 (58.5)	101 (61.2)	6 (33.3)	0.023
Nephropathy, No. (%)^a,b^	21 (50.0)	19 (50.0)	2 (50.0)	<0.9999
Anemia, No. (%)^a,b^	90 (19.5)	67 (16.7)	23 (38.3)	<0.0001
Neurologic disorders, No. (%)^a,b^	815 (98.9)	721 (99.2)	94 (96.9)	0.044
Motor disturbance, No. (%)^a,b^	265 (51.2)	230 (50.2)	35 (58.3)	0.237
Language impairment, No. (%)^a,b^	353 (72.5)	323 (74.8)	30 (54.5)	0.002
Abnormal neuropsychological test results, No. (%)^a,b^	263 (96.3)	235 (96.7)	28 (93.3)	0.679
Abnormal MRI results, No. (%)^a,b^	475 (93.0)	425 (93.2)	50 (90.9)	0.727
Hydrocephalus, No. (%)^a,b^	125 (24.5)	121 (26.5)	4 (7.3)	0.002

*No.* number, *IQR* interquartile range, *NBS* newborn screening, *MRI* magnetic resonance imaging.

^a^Cases for which the corresponding information or test results were not available are not counted in the totals used to calculate the percentages.

^b^Healty cases are not counted in the totals used to calculate the percentages.

**Table 2:** The biochemical results before and after treatment of patients in different groups.

	C3			C3/C2			uMMA			uMCA			Hcy		
	(0.40∼4.00 μmol/L)			(0.03∼0.20)			(0.0∼4.0 mmol/mol creatinine)			(0.0∼0.8 mmol/mol creatinine)			(0.0∼15 μmol/L)		
	Before treatment	After treatment	p1	Before treatment	After treatment	p1	Before treatment	After treatment	p1	Before treatment	After treatment	p1	Before treatment	After treatment	p1
**Overall**	7.13 (4.87–10.41)	3.43 (2.2–5.73)	<0.0001	0.6 (0.4–0.91)	0.14 (0.081–0.23)	<0.0001	114.3 (40.47–286.40)	7.2 (2.23–22.51)	<0.0001	3.13 (1.29–7.75)	0.79 (0–1.9)	0.0001	88.4 (51.09–148.9)	35.65 (23.6–48)	<0.0001
**Subtypes**															
cblC	6.68 (4.63–9.52)	3.11 (2.02–4.81)	<0.0001	0.58 (0.39–0.9)	0.12 (0.08–0.18)	<0.0001	95.25 (37.00–237.20)	5.6 (1.89–15.38)	<0.0001	2.7 (1.1–5.79)	0.63 (0–1.42)	<0.0001	102.3 (65–162)	36.5 (25–49)	<0.0001
mut	9.82 (6.34–15.48)	12.75 (4.79–24.46)	0.0089	0.64 (0.47–0.95)	0.52 (0.24–0.74)	<0.0001	246 (98.09–558.30)	116.6 (12.21–409.6)	<0.0001	6.85 (2.26–16.55)	2.93 (0.85–6.75)	<0.0001			
p2	<0.0001	<0.0001		0.027	<0.0001		<0.0001	<0.0001		<0.0001	<0.0001				
**NBS**															
Y(cblC)	6.78 (4.86–9.37)	2.70 (1.72–4.12)	<0.0001	0.57 (0.36–0.87)	0.1 (0.06–0.15)	<0.0001	82.80 (32.40–207.10)	4.35 (1.15–10.53)	<0.0001	2.6 (1.12–5.54)	0.44 (0–1.16)	<0.0001	105 (66.65–173.2)	33.53 (22.2–44.73)	<0.0001
N(cblC)	6.53 (4.46–9.66)	3.47 (2.38–5.21)	<0.0001	0.58 (0.39–0.91)	0.14 (0.1–0.21)	<0.0001	103.5 (36.29–258.40)	7.14 (2.78–20.00)	<0.0001	2.77 (1–5.92)	0.8 (0–1.67)	<0.0001	97.12 (63.28–146.7)	38.7 (26.6–52.9)	<0.0001
Y(mut)	7.49 (4.91–11.46)	6.68 (2.96–16.85)	0.78	0.57 (0.33–0.85)	0.30 (0.14–0.69)	<0.0001	127 (36.48–301.80)	27.79 (6.77–208.4)	<0.0001	4 (1.52–12.19)	1.6 (0.44–4.36)	<0.0001			
N(mut)	11.49 (7.73–18.51)	18.25 (8.35–27.14)	0.0046	0.71 (0.51–1.02)	0.59 (0.38–0.78)	<0.0001	321.50 (152.5–734.3)	211.90 (48.94–617.00)	0.0009	8.615 (3.69–19.48)	4.4 (1.475–9.93)	0.0008			
p3	>0.9999	<0.0001		>0.9999	<0.0001		0.43	<0.0001		>0.9999	<0.0001		0.16	<0.0001	
p4	<0.0001	<0.0001		0.0034	<0.0001		<0.0001	0.0003		0.037	0.0005				
p5	0.21	<0.0001		>0.9999	<0.0001		0.12	<0.0001		0.027	<0.0001				
p6	<0.0001	<0.0001		0.001	0.0004		<0.0001	0.0008		<0.0001	0.0007				

p1: the differences of the corresponding metabolites levels before and after treatment; p2: the differences of the corresponding metabolites levels between cblC group and mut group; p3: the differences of the corresponding metabolites levels in cblC patients between NBS group and non-NBS group; p4: the differences of the corresponding metabolites levels in mut patients between NBS group and non-NBS group; p5: the differences of the corresponding metabolites levels in NBS group between cblC patients and mut patients; p6: the differences of the corresponding metabolites levels in non-NBS group between cblC patients and mut patients.

*C3* propionylcarnitine, *C2* acetylcarnitine, *uMMA* urine methylmalonic acid, *uMCA* urine methylcitrate, *Y* yes, cblC or mut patients diagnosed by NBS; *N* no, cblC or mut patients diagnosed without NBS.

### Comparison of clinical features in cblC-MMA and mut-MMA patients

The clinical features of patients with two major MMA subtypes, cblC-MMA and mut-MMA, were first compared (Tables 1, 2). The median age of disease onset in the mut-MMA group was significantly younger than it was in the cblC-MMA group. As for the treatment, the VitB12-responsive rate was 100% in the cblC-MMA group, while it was only 46.3% in the mut-MMA group. For the prognosis, the two groups had a comparable proportion of good outcomes but a variable distribution of poor outcome manifestations. In patients with poor prognosis, the rate of death and anemia in the mut-MMA group was much higher, while the proportions of ocular complications and neurologic disorders including language impairment and hydrocephalus were statistically higher in the cblC-MMA group (Table 1). Biometabolically, the cblC-MMA group had lower blood C3, C3/C2 ratio, uMMA and uMCA before and after treatment compared to the mut-MMA group (Table 2). The above results show that mut-MMA has worse clinical symptoms and a worse prognosis than cblC-MMA.

### The influence of NBS on the clinical features of cblC-MMA and mut-MMA patients

Given that the clinical characteristics of individuals with cblC-MMA and mut-MMA differ, the effect of NBS on both groups was further evaluated (Tables 2, 3). The NBS group demonstrated a younger age at onset and lower proportions of pre-treatment onset and onset of symptoms when compared to the non-NBS group for both MMA subtypes (Table 3). Besides, NBS shortened the onset-to-treatment interval and decreased the early onset rate in cblC-MMA patients rather than mut-MMA patients. For prognosis, NBS reduced the proportions of death and anemia among cblC-MMA patients with poor prognosis and improved MRI results among mut-MMA patients (Table 3). In terms of characteristic metabolites, NBS significantly decreased levels of all markers after treatment in both subtypes, with mut-MMA NBS cases also showing lower pre-treatment levels (Table 2). The above indicates that NBS could improve the overall prognosis of MMA patients.

**Table 3:** Comparison of clinical characteristics between cblC-MMA and mut-MMA patients diagnosed by NBS.

Characteristics	cblC-MMA		p1	mut-MMA		p2
	NBS	non-NBS		NBS	non-NBS	
No.	558	742		112	174	
Male sex, No. (%)	298 (53.4)	425 (57.3)	0.18	56 (50.0)	104 (59.8)	0.13
Age during follow-up (years), median (IQR)	1.56 (0.79–2.80)	3.89 (1.82–6.72)	<0.0001	1.73 (0.78–3.32)	3.04 (1.42–6)	<0.0001
**Onset of disease**						
Onset age (months)	0.059 (0.01–0.21)	0.21 (0.051–0.67)	<0.0001	0.02 (0.01–0.25)	0.08 (0.01–0.98)	0.037
Onset of symptoms, No. (%)^a^	342 (62.3)	728 (98.6)	<0.0001	64 (58.7)	171 (98.3)	<0.0001
Early onset, No. (%)	212 (92.2)	504 (80.4)	<0.0001	42 (91.3)	30 (80.5)	0.14
**Treatment**						
Interval from onset to treatment (days)^b^	15 (1–37)	42 (12–167)	<0.0001	1.5 (−16 to 23)	16 (5–38)	0.39
Pre-treatment onset, No. (%)^a^	174 (43.4)	725 (98.9)	<0.0001	32 (37.7)	171 (98.3)	<0.0001
Responsive to vitB12 treatment, No. (%)^a^	527 (100.0)	644 (100.0)	NA	55 (56.1)	49 (38.9)	0.015
**Outcome**						
Good prognosis, No. (%)	287 (51.4)	80 (10.8)	<0.0001	59 (52.7)	23 (13.2)	<0.0001
Poor, No. (%)	271 (48.6)	662 (89.2)		53 (47.3)	151 (86.8)	
Deceased, No. (%)	10 (3.7)	53 (8.0)	0.025	12 (22.6)	55 (36.4)	0.095
Ocular complications, No. (%)^a,c^	33 (55)	67 (64.4)	0.31	1 (20.0)	5 (38.5)	0.85
Nephropathy, No. (%)^a,c^	4 (36.4)	15 (55.6)	0.48	0 (0.0)	2 (66.7)	>0.9999
Anemia, No. (%)^a,c^	34 (23.6)	32 (12.5)	0.0051	10 (47.6)	12 (31.6)	0.26
Neurologic disorders, No. (%)^a,c^	186 (98.9)	523 (99.4)	0.85	25 (100)	66 (97.1)	0.95
Motor disturbance, No. (%)^a,c^	43 (41)	180 (52.5)	0.051	10 (47.6)	23 (62.2)	0.42
Language impairment, No. (%)^a,c^	69 (71.9)	249 (75.5)	0.56	11 (68.8)	19 (50.0)	0.33
Abnormal neuropsychological test results, No. (%)^a,c^	61 (95.3)	174 (97.2)	0.75	9 (100.0)	18 (90.0)	0.85
Abnormal MRI results, No. (%)^a,c^	107 (93.0)	312 (93.2)	>0.9999	8 (66.7)	41 (97.6)	0.0068
Hydrocephalus, No. (%)^a,c^	25 (75.8)	93 (80.2)	0.76	1 (100)	3 (75)	>0.9999

p1: the differences of the corresponding items between cblC (NBS) group and cblC (non-NBS) group; p2: the differences of the corresponding items between mut (NBS) group and mut (non-NBS) group.

*No.* number, *IQR* interquartile range, *NBS* newborn screening, *MRI* magnetic resonance imaging.

^a^Cases for which the corresponding information or test results were not available are not counted in the totals used to calculate the percentages.

^b^Negative numbers represent days of treatment prior to onset.

^c^Healty cases are not counted in the totals used to calculate the percentages.

### Gene variants identified in cblC-MMA and mut-MMA patients

**Fig. 1:**
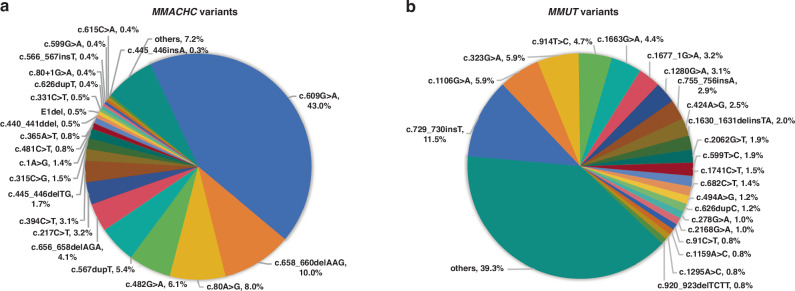
The variation spectrum of the *MMACHC* and *MMUT* genes in the cohort. Fan charts illustrating the *MMACHC* (**a**) and MMUT (**b**) variants and corresponding allele percentages in the cohort.

All patients involved in this study were ascertained by genetic analysis. Among 1319 cblC-MMA patients, 244 (18.5%) carried homozygous variants, 1051 (79.7%) carried compound heterozygous variants and 24 (1.8%) carried mono-heterozygous variants (Table S1). Totally 105 *MMACHC* variants were identified, including 35 missense, 21 nonsense, 6 splicing, 27 frameshift, 4 exon deletions, 12 small deletions and 28 variants were novel (Table S1). The five most frequent variants were c.609G > A (43.4%), c.658_660delAAG (10.0%), c.80A > G (8.0%), c.482G > A (6.1%) and c.567dupT (5.8%, Fig. 1 and Table S1), and the allele of c.609G > A was found in all top 5 most common combinations, another allele included c.609G > A (15.2%), c.658_660delAAG (9.9%), c.80A > G (8.7%), c.567dupT (5.2%) and c.217C > T (4.0%). Of the 298 mut-MMA patients, 16 (5.4%) carried homozygous variants, 276 (92.6%) carried compound heterozygous variants and 6 (2.0%) carried mono-heterozygous variants (Table S2). There were 159 variants identified in the *MMUT* gene, including 35 missense, 22 nonsense, 13 splicing, 19 frameshift, 2 exon deletions and 3 small deletions (Table S4). The c.729_730insTT (11.9%), c.1106G > A (6.0%), c.323G > A (6.0%), c.914T > C (4.8%) and c.1663G > A (4.1%) compromised the five most common variants (Fig. 1 and Table S2). The homozygous c.729_730insTT (2.0%), c.729_730insTT with c.1663G > A (2.0%), c.1106G > A (1.7%), c.323G > A (1.3%) and c.1106G > A with c.1663G > A (1.3%) were the top 5 common allele combinations.

### Factors affecting variable outcomes

Both cblC-MMA and mut-MMA patients were categorized into two groups based on whether they had poor outcomes and detailed abnormalities such as anemia, ocular complications, nephropathy, neurologic disorders (language impairment, motor disturbance, abnormal neuropsychological test results and abnormal MRI results) and death. Factors including “NBS”, “Pre-treatment onset”, “Responsive to VitB12 treatment”, biochemical metabolites at baseline and the top 5 most common variants in the *MMACHC* or *MMUT* genes. The differences in the incidence of these factors between poor outcome and control groups in patients with two subtypes are summarized in Tables S3–S6, and based on these, multivariate logistic regression was performed to explore factors independently associated with various prognostic phenotypes.

**Fig. 2:**
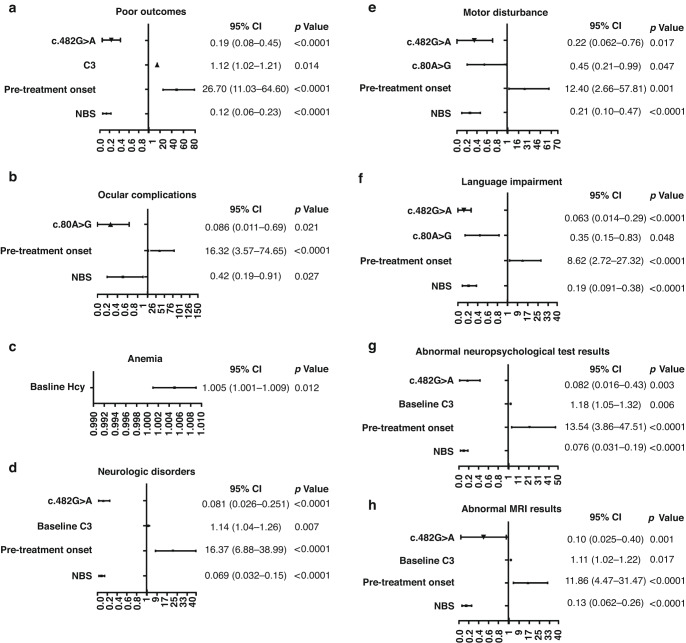
The effects of the potentially influencing factors on poor prognostic manifestations in cblC-MMA. The potentially predictor effects on overall poor outcomes (**a**), ocular complications (**b**), anemia (**c**), neurologic disorders (**d**), motor disturbance (**e**), language impairment (**f**), abnormal neuropsychological test results (**g**) and abnormal MRI results (**h**).

For cblC-MMA patients (Fig. 2), multivariate analysis revealed that “pre-treatment onset” (OR = 8.62–26.7) and “NBS” (OR = 0.03–0.42) were independent predictors of nearly all analyzed poor prognostic manifestations except death and anemia. A higher Hcy level was independently associated with a higher risk of anemia (OR = 1.01), and a higher baseline level of C3 could independently increase the risk of having an overall poor prognosis (OR = 1.11) and neurologic disorders (OR = 1.14), especially abnormal results of MRI (OR = 1.11) and neuropsychological tests (OR = 1.18). Moreover, the *MMACHC* hotspot variant of c.482G > A was a negative predictor of almost all poor prognostic phenotypes except anemia (OR = 0.06–0.22) and the variant of c.80A > G could independently decrease the risk of ocular complications (OR = 0.09), language impairment (OR = 0.35) and motor disturbance (OR = 0.45).

**Fig. 3:**
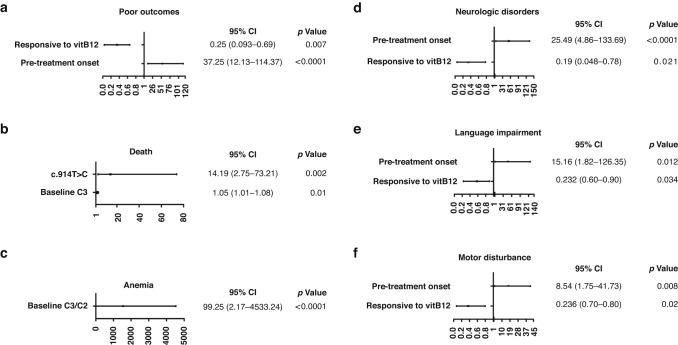
The effects of the potentially influencing factors on poor prognostic manifestations in mut-MMA. The potentially predictor effects on overall poor outcomes (**a**), death (**b**), anemia (**c**), neurologic disorders (**d**), language impairment (**e**) and motor disturbance (**f**).

For mut-MMA patients (Fig. 3), “pre-treatment onset” (OR = 8.54–37.25) and “responsive to VitB12 treatment” (OR = 0.19–0.25) were independent predictors of overall poor prognosis and neurologic disorders (especially language impairment and motor disturbance). Higher levels of baseline C3 and C3/C2 independently predicted a higher risk of death (OR = 1.05) and anemia (OR = 99.3), respectively. Moreover, the *MMUT* hotspot variant of c.914T > C (OR = 14.2) was a significant predictor of death.

### Metabolite levels after treatment and their diagnostic value for prognostic manifestations

**Fig. 4:**
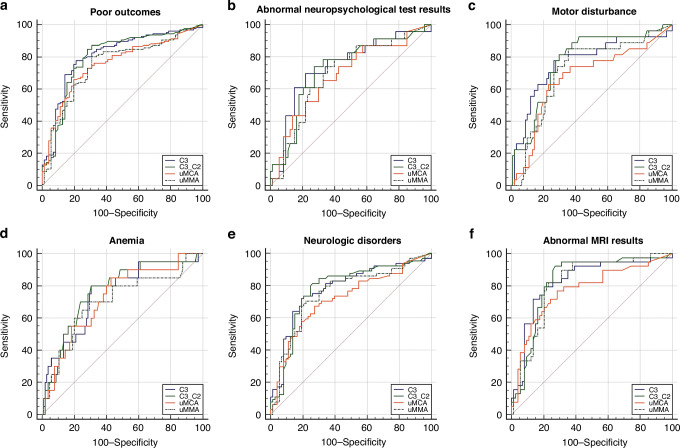
The diagnostic value of metabolite levels after treatment for poor outcomes in mut-MMA. ROC curve of four diagnostic metabolites for the diagnosis of poor outcomes (**a**), abnormal neuropsychological test results (**b**), motor disturbance (**c**), anemia (**d**), neurologic disorders (**e**) and abnormal MRI results (**f**) in mut-MMA.

Levels of prognostic metabolite indicators can assess the extent of recovery after treatment. Generally, patients with poor prognosis had significantly higher levels of prognostic metabolite markers when compared to patients without corresponding symptoms, and the mut-MMA group had higher marker levels than the cblC-MMA group (Table S4). However, for some poor outcomes like death, anemia and ocular complications, there was little difference in indicator levels between the groups with and without poor phenotypes for both MMA subtypes. Moreover, ROC curves illustrated that levels of all four characteristic metabolites after treatment had moderate diagnostic value for various poor outcomes in mut-MMA patients, such as C3 for overall poor prognosis (area under the curve [AUC] = 0.763, Fig. 4a); C3/C2 and C3 for abnormal neuropsychological test results (AUC = 0.722 and 0.737, Fig. 4b); uMMA, C3 and C3/C2 for motor disturbance (AUC = 0.714, 0.766 and 0.772, Fig. 4c); and uMCA, uMMA, C3 and C3/C2 for anemia (AUC = 0.726, 0.704, 0.750 and 0.768, Fig. 4d), neurologic disorders (AUC value = 0.718, 0.755, 0.780 and 0.779, Fig. 4e) and abnormal MRI results (AUC value = 0.772, 0.815, 0.827 and 0.829, Fig. 4f). However, none of the prognostic metabolic indicators has diagnostic value for poor prognosis in cblC-MMA patients (AUC < 0.7).

## Discussion

The long-term prognosis of MMA patients varies from normal to life-threatening and is influenced by multiple factors. In this national multicenter retrospective study with one of the largest Chinese MMA cohorts, we provided rich and reliable information on prognostic manifestations and their possible influence of the two major subtypes in combined and isolated MMA, cblC and mut, in mainland China.

The Chinese MMA population exhibits distinct characteristics, mainly due to the different subtype distribution and genetic variation spectrum compared to studies from America and Europe. In contrast to many Western countries where MMA is predominantly of the isolated type, the combined type accounts for 60%–80% of cases in China.^35, 36^ Furthermore, the variation hotspots of *MMACHC* and *MMUT* also differ greatly between China and other countries. For instance, the most prevalent *MMACHC* variants were c.609G > A in China,^37^ while c.271dupA and c.331C > T occurred predominantly in European and American populations and c.394C > T was frequent in patients from the Middle East and India.^38, 39^ The c.729_730insTT in the *MMUT* gene had the highest frequency in our study, which contrasts with variants like c.322C > T in Spanish, c.349G > T in Japanese and c.655A > T in French.^40^ It is noteworthy that the incidence of MMA in China exceeds the global average. A detailed analysis of the prognosis of MMA patients in China will help to make more specific recommendations for the management of our patients.

The MMA prognosis depends largely on the subtypes. Despite similar poor prognosis rates of around 70%, mut-MMA patients have a markedly higher mortality rate and higher levels of all four MMA biochemical indicators after treatment when compared to cblC-MMA patients. These findings indicated a worse outcome in the mut-MMA group, which may be attributed to the more frequent episodes of acute metabolic decompensation, earlier onset and lower VitB12-responsive rate of mut-MMA patients (Table 1).^41–43^ Furthermore, the “pre-treatment onset” and unresponsiveness to VitB12 treatment were also independently associated with poor prognosis and neurologic disorders in mut-MMA patients, as evidenced by our multivariate analysis and previous studies.^44^ Moreover, elevated baseline levels of C3 and C3/C2 were predicted as independent positive predictors of death and anemia in mut-MMA patients, respectively. In MMA, a significant quantity of propionyl-CoA is unable to be converted to succinyl-CoA to enter the TCA cycle due to enzyme defects and needs to be combined with carnitine to form C3 for excretion. Therefore, elevated C3 can be an indicator of abnormal propionate catabolism. C3/C2 were considered a more specific metabolic indicator than C3. Consequently, abnormal propionate metabolism was highly correlated with death and anemia observed in mut patients, which requires strict monitoring and control. Finally, the c.914T > C variant in the *MMUT* gene, specific to the Chinese population, was identified as an independent risk factor for mortality, emphasizing the importance of rigorous management in affected patients.

For cblC-MMA prognosis, a higher incidence of language impairment, ocular complications and hydrocephalus among survivors with poor outcomes was observed compared to those with mut-MMA, with the latter two having been previously reported.^45, 46^ This underscores the importance of ophthalmological, MRI, and language assessments during follow-up for cblC-MMA patients. With regard to the factors affecting the prognostic manifestations, our findings showed that higher baseline C3 levels increased the risk of poor prognosis and neurologic disorders, suggesting that the severe suppression of the propionate metabolic pathway is prone to irreversible nerve damage. The baseline Hcy levels independently predicted anemia. This discovery is supported by previous studies demonstrating the prevalence of megaloblastic anemia in patients with the cblC type, which is associated with hyperhomocysteine.^47, 48^ In addition, variant hotspots c.80A > G and c.482G > A in the *MMACHC* gene, mainly found in patients with late-onset and mild phenotypes, were identified to be strongly related to a better prognosis.^49, 50^

Similar to the findings of mut-MMA, pre-treatment onset predicted most manifestations of poor outcomes for cblC-MMA, highlighting the irreversible damage of MMA onset and underscoring the urgency of timely diagnosis and treatment for prognostic recovery. NBS holds potential for early detection and intervention in rare, treatable illnesses and its function in improving the prognosis of patients with cblC-MMA was confirmed by prior studies and our multivariate analysis.^23, 25^ In contrast, no poor outcomes were identified to be independently associated with NBS in mut-MMA, possibly due to the fact that more mut-MMA patients develop symptoms before or during the process of NBS, a more severe condition with limited therapeutic effect, as shown in our study (Table 1). Nevertheless, NBS can facilitate early treatment and significantly reduce the symptom onset in mut patients, and the lower metabolic indicator levels before treatment in the NBS group of mut-MMA patients indicated that the early diagnosis due to NBS can effectively reduce the occurrence of damaging or even fatal acute metabolic derangement at initial presentation. Therefore, the importance of NBS for the diagnosis, treatment and prognosis of mut-MMA cannot be ignored, and the screening window needs to be as early as possible. Notably, in contrast to studies based on Chinese populations, the benefit of NBS on MMA outcomes is less evident in some Western countries.^51, 52^ This discrepancy may be attributed to the fact that Western countries have a greater proportion of patients with cobalamin-nonresponsive MMA and a different spectrum of genetic variants.^35^ In conclusion, NBS for MMA is necessary for China, which has a large proportion of a cobalamin-responsive MMA population.

In addition to the factors mentioned above, significantly elevated baseline uMMA and uMCA levels, higher proportions of patients with the *MMACHC* variants c.609G > A, c.80A > G or c.658_660delAAG, or with the *MMUT* variant c.323G > A and a lower percentage of patients with the *MMUT* variant c.1663G > A were found in some poor outcome groups compared to those without corresponding manifestations (Table 2 and Tables S4–S6). The elevated MMA level has been linked to increased risk for mortality and basal ganglia stroke,^53, 54^ and MCA could contribute to brain damage through impairing glutamate metabolism and mitochondria.^55^ Moreover, the genotype–phenotype associations have also been reported for some of the above variants: irreversible brain disorders and poor prognosis were more common in cblC patients with the *MMACHC* c.609G > A homozygous variant; the *MMACHC* c.80A > G variant was prevalent among Chinese cblC patients with prominent renal complications; and the c.1663G > A variant in *MMUT* is associated with better outcomes in mut-MMA.^7, 56, 57^ However, the multivariate analysis in our study identified no strong association between these elements and any poor prognostic manifestations. We must recognize that the exclusions in multivariate analysis due to missing information might introduce bias. The associations of MMA prognosis with these elements still need to be further explored based on an intact and large patient database.

Biometabolic indicators can reflect an individual’s response to a therapeutic intervention and the prognosis of a disease.^58^ In line with this, significant differences in the characteristic metabolite levels after treatment were found between the groups with and without corresponding prognostic symptoms, especially for neurologic disorders (Table 2 and Table S4). The distinctions were more pronounced for mut-MMA, evident in the diagnostic value of the four indicators for prognostic symptoms (Fig. 4), which are meaningful for prognostic surveillance and management. However, for cblC-MMA patients and symptoms like death, ocular complications and anemia, the differences and diagnostic value were inconclusive. The onset of symptoms in these cases may involve a combination of metabolites or other factors, which need further research.

## Conclusions

The study outlines the long-term prognosis of a large MMA cohort. The comparative study of the clinical features between two major MMA subtypes enhances understanding of the phenotypic and prognostic variations of this disease. Our analysis provided strong evidence of the clinical effectiveness and long-term benefits of MS/MS-based NBS for MMA. It is crucial to expand NBS coverage and optimize the screening window. There are limitations to incomplete data collection, which affected the conduct and accuracy of some statistical analysis, and the prognostic manifestations involve other systems such as blood and viscera that need further attention. Nonetheless, the identified influential factors will refine diagnosis and treatment strategies, improving the MMA prognosis.

## Supplementary information

Supplementary information

Supplementary information

## Data Availability

Data will be made available on request.
