# Supplementary material for: Long-term follow-up of Chinese patients with methylmalonic acidemia of the cblC and mut subtypes
Source: Pediatr Res. 2024 Sep 21;97(6):2010–9. doi: 10.1038/s41390-024-03581-x (PMC12122358; doi:10.1038/s41390-024-03581-x)

SUPPLEMENTAL MATERIAL

Fig. S1 The prognosis of the patients in the cohort. Summary of the NBS status, disease onset, treatment and clinical outcome for the patients with cblC-MMA (left) and mut-MMA (right) in this study.

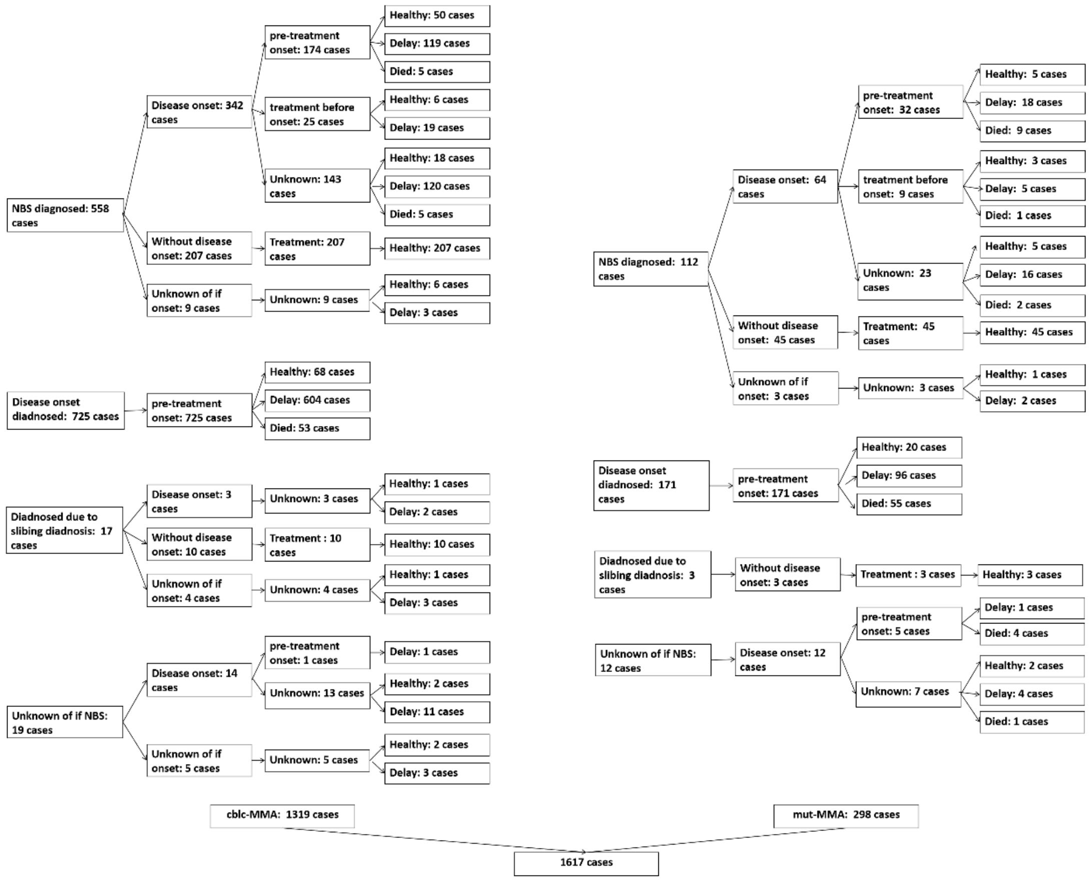

Supplement: Supplementary file 2 — Supplementary information [file 41390_2024_3581_MOESM2_ESM.pdf]
